# Antibacterial activity of *Aquilaria crassna* leaf extract against *Staphylococcus epidermidis* by disruption of cell wall

**DOI:** 10.1186/1476-0711-12-20

**Published:** 2013-08-20

**Authors:** Sirilak Kamonwannasit, Nawarat Nantapong, Pakarang Kumkrai, Prathan Luecha, Sajeera Kupittayanant, Nuannoi Chudapongse

**Affiliations:** 1 School of Pharmacology, Institute of Science, Suranaree University of Technology, Nakhon Ratchasima 30000, Thailand; 2 School of Microbiology, Institute of Science, Suranaree University of Technology, Nakhon Ratchasima 30000, Thailand; 3 Department of Pharmacognosy and Toxicology, Faculty of Pharmaceutical Sciences, Khon Kaen University, Khon Kaen 40002, Thailand; 4 School of Physiology, Institute of Science, Suranaree University of Technology, Nakhon Ratchasima 30000, Thailand

**Keywords:** *Aquilaria crassna*, *Staphylococcus epidermidis*, Antioxidant, Anitbacterials, Cell wall, Acute toxicity

## Abstract

**Background:**

*Aquilaria crassna* Pierre ex Lecomte has been traditionally used in Thailand for treatment of infectious diseases such as diarrhoea and skin diseases for a long time. The main objectives of this study were to examine antibacterial activity of the *Aquilaria crassna* leaf extract against *Staphylococcus epidermidis* and its underlying mechanism. The antioxidant activity and acute toxicity were studied as well.

**Methods:**

Antioxidant activities were examined by FRAP, ABTS and DPPH scavenging methods. Antibacterial activity was conducted using disc diffusion assay and the minimum inhibitory concentration (MIC) was determined by dilution method. The minimum bactericidal concentration (MBC) was reported as the lowest concentration producing no growth of microbes in the subcultures. Morphological changes of the microbe were observed by scanning electron microscopy, while an inhibitory effect on biofilm formation was evaluated by phase contrast microscopic analysis. Bacterial cell wall integrity was assessed by transmission electron microscopy. Acute toxicity was conducted in accordance with the OECD for Testing of Chemicals (2001) guidelines.

**Results:**

The extract exhibited considerable antioxidant activity. *Staphylococcus epidermidis* was susceptible to the extract with the MIC and MBC of 6 and 12 mg/ml, respectively. The extract caused swelling and distortion of bacterial cells and inhibited bacterial biofilm formation. Rupture of bacterial cell wall occurred after treated with the extract for 24 h. Acute toxicity test in mice showed no sign of toxicity or death at the doses of 2,000 and 15,000 mg/kg body weight.

**Conclusion:**

The aqueous extract of *Aquilaria crassna* leaves possesses an *in vitro* antibacterial activity against *Staphylococcus epidermidis*, with no sign of acute oral toxicity in mice, probably by interfering with bacterial cell wall synthesis and inhibiting biofilm formation.

## Introduction

Agarwood is mainly produced by trees in the species of *Aquilaria* (family Thymelaeaceae). These plants provide economically important natural products which are used for the production of incense, perfumes and traditional medicines in Asia [1]. At least four species of agarwood trees are found in tropical rainforest areas of Thailand, namely *Aquilaria crassna* Pierre ex Lecomte, *A. subintegra, A. malaccensis*, and *A. rugosa*[2]. Owing to over-exploitation, these plant species are nowadays considered as endangered species of Southeast Asia. Therefore, cultivation of agarwood is encouraged to reduce the harvest from wild populations. Since it takes about 10 years before the wood can produce valuable essential oil or resin, leaves from young agarwood are collected for production of healthy tea in Viet Nam, Combodia and Thailand.

The agarwood extract has been used as one of the active ingredients in several Thai traditional pharmaceutical preparations, such as “Krisanaglun” which is used as antispasmodic, antidiarrheal agent and cardiovascular function enhancer in fainted patient. Based on Thai folklore information, several parts of agarwood have been used for a long time in the treatment of infectious diseases such as diarrhoea, dysentery and skin diseases. Recently, the antibacterial activities of *A. crassna* leaf extract against enteric bacteria, such as *Staphylococcus aureus*, *Clostoridium difficile*, *Peptostreptococcus anaerobius* and *Bacteroides fragilis*, have been reported [3]. However, its inhibitory effect on *S. epidermidis*, the pathogen known to cause skin disease and one of the most important opportunistic pathogens, has never been documented. In the present study, the antibacterial activity against *S. epidermidis* of the aqueous extract of *A. crassna* leaves and possible mechanism were investigated. The phytoconstiuents, antioxidant properties and acute toxicity of the extract were studied as well.

### Experimental procedures

#### Plant material

*A. crassna* leaves were collected from a cultivated field in Nakhon Ratchasima province, Thailand. The plant was identified by a botanist, Dr. Paul J. Grote, School of Biology, Suranaree University of Technology (SUT) and specimen of the plant has been kept at School of Pharmacology, SUT. The voucher specimen number is Pharm-Chu-005.

The leaves were oven dried at 50°C, and then cut into small pieces. Dried leaves (24 g) were extracted in boiling water (400 ml) for 30 min twice. The pooled extracts were filtered and concentrated at 40°C using a rotary evaporator under low pressure. The residue was freeze-dried in a lyophilizer. The extract with a total yield of 14.2% was stored at −20°C until used.

### Phytochemical screening

Phytochemical screening procedures were carried out according to the standard methods previously reported [4,5]. Qualitative phytochemical compositions of the crude extract of *Aquilaria crassna* leaves were determined for the presence of alkaloids, flavonoids, tannins, saponins and cardiac glycosides.

### Determination of total phenolic compounds

The amount of total phenolic compounds was measured by a method described by Matthaus [6]. In brief, 5 mg of the extract was dissolved in 1 ml of distilled water. A 100 μl aliquot of this mixture was added to 2 ml of 2% Na_2_CO_3_ followed by 100 μl of Folin-Ciocalteau reagent in methanol (1:1 v/v). After 30 min of incubation, the absorbance was measured at 750 nm. The concentration was calculated using gallic acid as a standard. The results were expressed as milligrams gallic acid equivalents (GAE) per gram extract.

### Determination of antioxidant activity

#### Scavenging effects on DPPH radicals

To measure antioxidant activity, the 2,2-diphenyl-1-picrylhydrazyl hydrate (DPPH) radical scavenging assay was carried out according to the procedure described previously [7]. The crude extract (100 μl; final concentration range from 0–50 μg/ml) was added to 4.0 ml of 50 μM DPPH in methanolic solution and the final volume was adjusted to 5.0 ml with water. After vortexing, the mixture was incubated for 30 min in the dark at room temperature. The decrease in absorbance at 517 nm was measured using a spectrophotometer. Antioxidant activity was expressed as IC_50_, which was defined as the concentration of the extract required to inhibit the formation of DPPH radicals by 50%.

### ABTS assay

ABTS (*2,2'-azino-bis(3-ethylbenzthiazoline-6-sulphonic acid)* radical-scavenging activity of extract was carried out according to the procedure described previously [8]. ABTS radical cation (ABTS^•+^) was produced by the reaction between 5 ml of 14 mM ABTS and 5 ml of 4.9 mM potassium persulfate (K_2_S_2_O_8_). The resulting solution was stored in the dark at room temperature for 16 h. Before used, the solution was diluted with ethanol to give an absorbance of 0.700 ± 0.020 at 734 nm. The plant extract (50 μl) at various concentrations were added to 950 μl of ABTS solution and mixed thoroughly. The reaction mixture was allowed to stand at room temperature for 6 min, the absorbance was measured at 734 nm and compared to the standard butylated hydroxytoluene (BHT).

### Ferric reducing antioxidant power (FRAP) assay

The FRAP assay was conducted according to procedure described by Dordevic *et al.*[9] with minor modification. The FRAP reagent consists of 10 mM TPTZ (2,4,6-tripyridyl-striazine) in 40 mM HCl, 20 mM FeCl_3_, and 300 mM acetate buffer (pH 3.6) in proportions of 1:1:10 (v/v/v). Fifty μl of the sample was added to 1.5 ml of FRAP reagent (freshly prepared and warmed to 37°C before used). The absorbance was measured at 593 nm using a UV spectrophotometer after 4 min of incubation. A standard curve was constructed using FeSO_4_ solution. The results were expressed as μmol Fe^2+^/mg dry weight of plant material. All measurements were carried out in triplicate and the mean values were calculated.

### Antibacterial assays

#### Disc diffusion assay

The antibacterial activity of the crude extract was assayed against *S. epidermidis* (obtained from Thailand Institute of Scientific and Technological Research; TISTR 518) using disc diffusion method previously described [10]. Briefly, 100 μl of bacteria (10^8^ CFU/ml) was spread onto the Mueller-Hinton agar plate. The extract (2, 4 and 6 mg) was applied to filter paper discs (Whatman No. 1, 6 mm diameter) and then placed on the previously inoculated agar plate. After 24 h of incubation at 37°C, clear inhibition zones around the discs indicated the presence of antibacterial activity. The assay was carried out in triplicates. Vancomycin (30 μg) was used as a positive control.

### Determination of minimum inhibitory concentration (MIC) and minimum bactericidal concentration (MBC)

Determination of the minimum inhibitory concentration (MIC) against *S. epidermidis* (10^4^ CFU/ml) was conducted by a two-fold serial dilution method in Mueller-Hinton broth (MHB). MIC was considered as the lowest concentrations of the agents that yielded no visible growth of microorganisms after 24 h of incubation at 37°C. The MBC determination was carried out by subculturing 100 μl from each tube from the MIC assay onto fresh substance-free MH agar plates. The MBC was defined as the lowest concentration of agent that produced no growth of subcultures.

### Biofilm assay

The inhibitory effect of the extract on biofilm formation was examined by microscopic analysis. A culture of *S. epidermidis* was prepared in tryptic soy broth (TSB) at 37°C for 24 h. 30 μl aliquots of the culture were pipetted into each well of 24-well plates in the presence of 3 ml TSB and incubated at 37°C for 24 h to form biofilm. Thereafter, medium was replaced with fresh medium containing the extract of *A. crassna* leaves or vancomycin. After incubation of another 24 h, the medium was removed and each well was gently washed three times with phosphate buffer solution. The inhibitory effect on biofilm formation was observed by phase contrast microscopy.

### Scanning electron microscopy

Scanning electron microscopy (SEM) was performed on *S. epidermidis* treated with MIC of *A. crassna* leaf extract. *S. epidermidis* was cultured to reach mid-log phase in MHB before use. Control and treated cells were prepared for morphological observation. The bacterial samples were washed five times with fresh media and then fixed with 2.5% glutaraldehyde in phosphate buffer (pH 7.2) at 4°C for 1 h, washed three times with phosphate buffer for 10 min and fixed with 1% osmium tetroxide for 2 h. This was followed by three washings in phosphate buffer for 10 min and subsequently dehydrated in a series of ethanol concentrations (30%, 50%, 70%, 90% and 95%), for 15 min each. The samples were subjected to 100% ethanol and CO_2_ to achieve the critical point and then coated with gold ion in a pressure metallic chamber. At the end of the process, the samples were submitted for analysis by SEM.

### Transmission electron microscopy

Cellular damage of bacteria was examined using transmission electron microscopy (TEM). Bacterial cells treated with vehicle, vancomycin and the extract of *A. crassna* leaves were harvested after 24 h of incubation and fixed in 2.5% glutaraldehyde in 0.1 M phosphate buffer, for 2 h. The cells were washed three times with 0.05 M phosphate buffer (pH 7.2) and postfixed for 2 h with 1% osmium tetroxide in 0.1 M phosphate buffer (pH 7.2) at room temperature. After washed twice in phosphate buffer, the cells were dehydrated through serial graded concentrations of ethanol (35, 70, 95 and 100%, respectively) for 15 min, then infiltrated and embedded in Spurr’s resin. Ultrathin sections were cut with a diamond knife using an ultramicrotome and then mounted on bare copper grids. Finally, specimens were counterstained with 2% (w/v) for 3 min and then with 0.25% (w/v) lead citrate solution for 2 min and examined with Tecnai G2 electron microscope (FEI, USA) operated at 120 kV.

### Acute toxicity in mice

The acute toxicity test performed in this experiment was conducted by following the OECD guidelines for Testing of Chemicals (2001) [11]. The aqueous extract of *A. crassna* leaves was prepared in the concentrations of 200 mg/ml and 750 mg/ml by dissolving in distilled water for dosing group of 2,000 and 15,000 mg/kg body weight respectively. All mice were monitored for clinical signs of toxicity at 0.5, 1 and 3 h after oral administration of the extract and once daily thereafter for 14 days. The organs of animals were removed immediately after sacrificed on day 14 and then examined macroscopically for pathological changes.

### Statistical analysis

All experimental results were expressed as means ± standard deviation. The differences among groups in the acute toxicity test were analyzed by one way ANOVA followed by Student-Newman-Keuls test. The results with *p* value < 0.05 were considered as statistically significant differences.

## Results

### Phytochemical screening, phenolic content and antioxidant activity

Phytochemical analysis of the extract indicated the presence of flavonoids, tannins, saponins, cardiac glycosides but the absence of alkaloids. Total phenolic content was 176.61 ± 24.46 mg GAE/g extract (n=3). The results of radical scavenging capacities which were determined by ABTS, FRAP and DPPH scavenging methods are shown in Table 1. The IC_50_ of *A. crassna* leaf extract to scavenge DPPH radical was 7.25 ± 2.05 μg/ml whereas that of ascorbic acid was 1.33 ± 0.08 μg/ml. The IC_50_ values of ABTS radical scavenging activity of the extract and the standard BHT were 218.93 ± 29.77 μg/ml and 83.09 ± 0.45 μg/ml, respectively. The FRAP value of the extract was 1.18 ± 0.07 μmol of Fe^2+^/mg dried extract.

**Table 1:** **Radical scavenging activities and reducing power of the aqueous extract of *A. crassna *leaves**

	**Methods**		
	**DPPH radical**	**ABTS**	**FRAP**
	**(IC**_**50**_**: μg/ml)**	**(IC**_**50**_**: μg/ml)**	**(μmol Fe**^**2+**^**/mg dried extract)**
The *A. crassna* leaf extract	7.25 ± 29.77	218.93 ± 29.77	1.18 ± 0.07
Ascorbic acid	1.33 ± 0.08	**-**	**-**
Butylated hydroxytoluene (BHT)	-	83.09 ± 0.45	**-**

Values are expressed as means ± standard deviation (n=3).

### Antibacterial activity

By disc diffusion assay, it was found that the extract inhibited growth of *Staphylococcus epidermidis* (Table 2) at 2, 4 and 6 mg. Vancomycin was employed as a positive control. By macro-dilution method, the MIC and MBC of the extract was 6 and 12 mg/ml, respectively.

**Table 2:** **Antibacterial activity of the aqueous extract of *A. crassna *leaves against *S. epidermidis***

**Antibacterial activity**	**Agents**			
	**The *A. crassna *leaf extract**			**Vancomycin 30 μg**
	**2 mg**	**4 mg**	**6 mg**	
Diameters of inhibition zone (mm)	12.0 ±1.0	15.0 ± 0.4	18.0 ± 1.0	21.0 ± 1.0
MIC		6 mg/ml		1.5 μg/ml
MBC		12 mg/ml		3.0 μg/ml

Values are expressed as means ± standard deviation (n=3).

### Effects of the extract on biofilm formation

**Figure 1:**
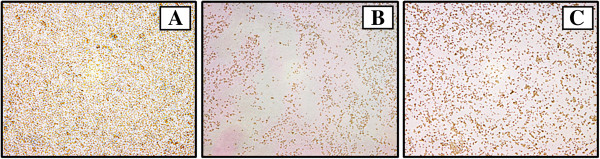
**Microscopic images on biofilm formation of *S. epidermidis *treated with *A. crassna *leaf extract.** Cells treated with vancomycin **(B)** or the extract **(C)** formed less biofilm compared to control sample **(A)**. Enlargement: ×400.

Crystal violet staining of surface-attached cells by microtiter plate assay is a popular and convenient method for quantitative detection of bacterial biofilm formation; however direct microscopic observation is strongly recommended to confirm the data from staining analysis [12]. Due to the absorption of the *Aquilaria crassna* leaf extract at 595 nm, in the present study the effect on biofilm formation was investigated by microscopic analysis which gave more accurate imformation. The phase contrast microscopic images (Figure 1) showed that *Staphylococcus epidermidis* formed vast empty regions on surface with a few clusters of cells and scattering of single cell when treated with the extract and vancomycin.

### Morphological changes and rupture of bacterial cell wall induced by the extract

**Figure 2:**
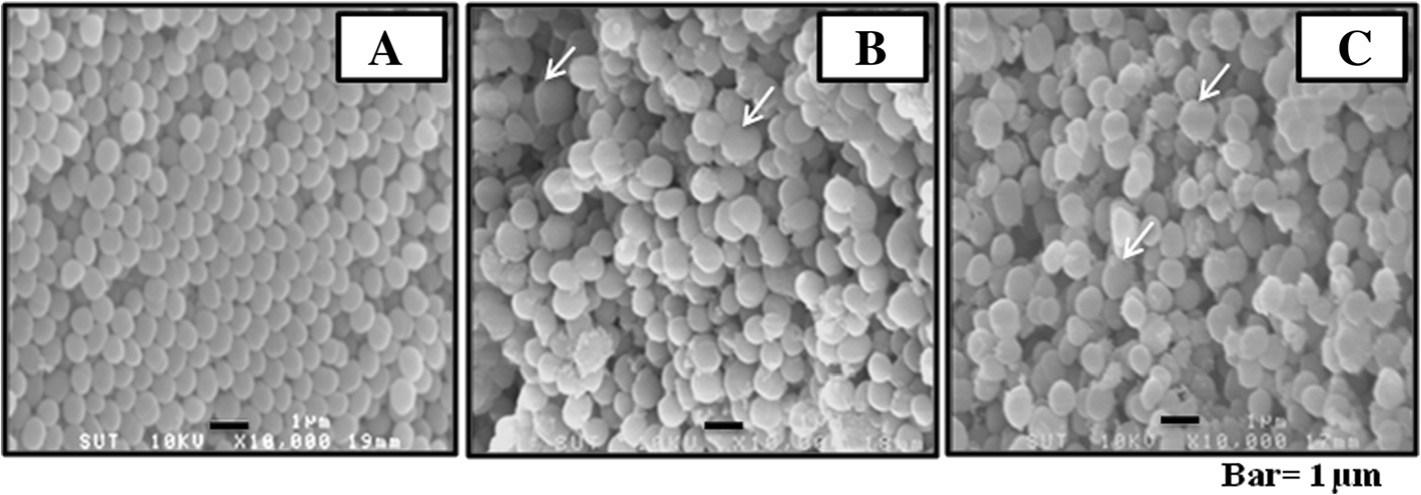
**Scanning electron micrograph of *S. epidermidis *treated with *A. crassna *leaves extract.** Cells were treated as described in Material and Method. The swollen cells (arrows) were observed after treated for 24 h with vancomycin **(B)** and the extract **(C)**, compared with regular shape of control **(A)**. Enlargement: ×10000.

**Figure 3:**
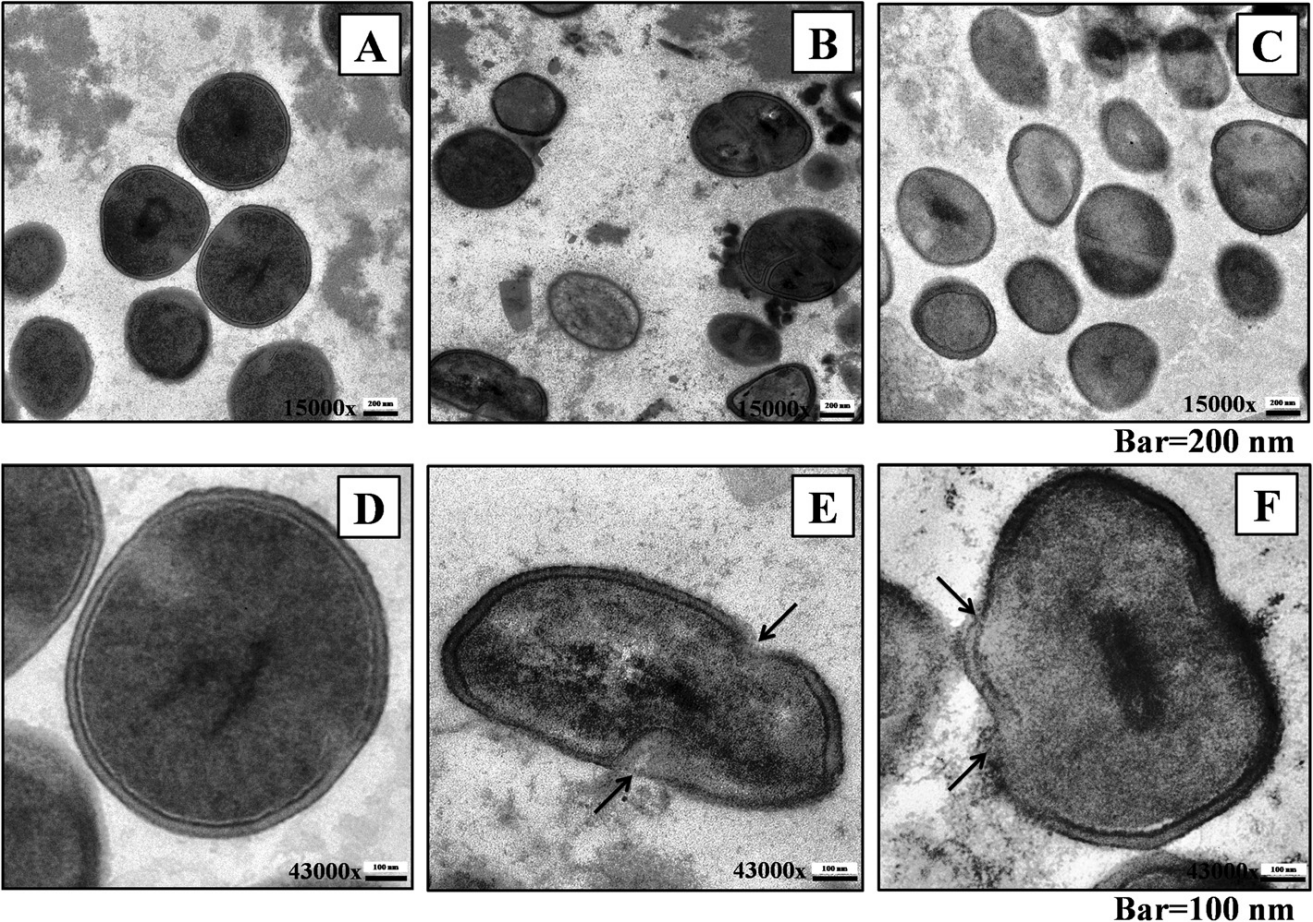
**Transmission electron micrograph of *S. epidermidis *treated with *A. crassna *leaves extract. A**, **B** and **C** are overview of the untreated cells and cells treated with vancomycin and the extract, respectively (Enlargement: ×15000). Bacterial cells with irregular shape were seen in both treated groups. Damaged cell walls (arrows) were observed after 24 h of incubation with vancomycin **(E)** and the extract **(F)**, compared with control **(D)**. Enlargement: ×43000.

*Staphylococcus epidermidis* was treated with the extract at MIC (6 mg/ml) and incubated at 37°C for 24 and 48 h. SEM analyses were performed and compared to untreated and vancomycin-treated groups. Control bacteria in the absence of the extract showed regular morphology (Figure 2A), whereas cells treated with vancomycin (Figure 2B) and the aqueous extract of *A. crassna* leaves (Figure 2C) appeared swelling and distorting after 24 h of incubation. TEM monographs showed that vancomycin and the extract caused rupture of bacterial cell wall and alteration of bacterial shape compared to control (Figure 3).

### Acute oral toxicity of *Aquilaria crassna* leaf extract in mice

The acute toxicity of aqueous *Aquilaria crassna* leaf extract in mice is summarized in Table 3. The results showed that with the doses of 2,000 and 15,000 mg/kg body weight of the extract, all treated mice did not exhibit abnormal signs of toxicity or deaths. Normal increase in body weight and none of gross pathological lesions were observed on the control and treated mice.

**Table 3:** **Acute oral toxicity of the extract of *A. crassna *leaves in mice**

**Treatment (mg/kg)**	**sex**	**Body weight (g)**			**Mortality**	**Symtoms of toxicity**	**Gross pathology**
		**Day 1**	**Day 8**	**Day 15**			
0		33.20 ± 1.52	37.06 ± 1.52	41.00 ± 1.73	0	None	Normal
2000	Male	32.80 ± 1.30	37.20 ± 0.84	41.00 ± 1.00	0	None	Normal
15000		34.40 ± 1.14	39.80 ± 0.84	43.20 ± 0.84	0	None	Normal
0		28.40 ± 1.67	30.80 ± 2.17	34.00 ± 1.58	0	None	Normal
2000	Female	28.00 ± 1.22	30.00 ± 1.22	33.2 ± 1.64	0	None	Normal
15000		28.80 ± 1.30	31.80 ± 1.30	34.00 ± 1.00	0	None	Normal

Value are expressed as means ± standard deviation (n = 5); *p*> 0.05 compared to control.

## Discussion

There are several lines of evidence showing positive correlation between total phenolic content and antioxidant properties [13-15]. In addition to antioxidant activity, plant phenolic compounds, such as flavonoids and tannins, have been shown to possess antimicrobial activities [16-18]. ABTS, DPPH and FRAP assays are widely used to determine the antioxidant capacity in plant extracts because of their simplicity, stability and accuracy [19]. As shown in Table 1, our results showed that the aqueous extract of *A. crassna* leaves exhibited substantial antioxidant activity, suggesting its potential to possess antimicrobital activity. Recently, a group of researchers from Japan has reported antibacterial activities of the aqueous extract of *A. crassna* leaves against enteric bacteria, such as *S. aureus*, *Clostoridium difficile*, *Peptostreptococcus anaerobius* and *Bacteroides fragilis*[3]. Herein, we tested its inhibitory effect on the Gram-positive bacteria using *S. epidermidis* TISTR 518. This type of microorganism is one of coagulase-negative staphylococci which become increasingly recognized as pathogen of nosocomial infection, following ophthalmologic, neurologic and cardiothoracic surgery, in immunocompromised patients and in patients with prosthetic devices. This microbe was found susceptible to the extract with MIC of 6 mg/ml, which is in the range of MICs (4–8 mg/ml) against other bacterial strains reported by Kakino and colleagues [3]. We further investigated and found that the extract exhibited bactericidal activity with MBC of 12 mg/ml.

The ability to form biofilm, a slimy layer with embedded microcolonies, is one of the most important and one of the most widespread virulence factors occurring in microbes. Biofilms grows easily on surfaces of artificial materials used for catheters and prosthetic devices [20]; and it is estimated that biofilms are associated with about 65% of nosocomial infections. It has been suggested that biofilm formation is the main virulence mechanism of *S. epidermidis*[21]. In addition to growth inhibitory effect, the extract was found to impede the production of slimy biofilm by *S. epidermidis* similar to vancomycin which has been shown to inhibit *S. epidermidis* biofilm formation [22].

It is widely accepted that plants are good sources of novel antimicrobial agents. Screening of antimicrobial activities to find which type of bacteria are susceptible to plant extracts is useful, however the investigation of underlying mechanism is also crucial for drug development. To explore the possible antibacterial mechanism, we studied the effect of the extract on morphological changes of *S. epidermidis* cells by SEM and TEM. Vancomycin, an antibiotic which is widely known to damage bacterial cell wall of most Gram-positive bacteria, including staphylococci and enterococci, was used as positive control. As revealed by SEM, bacterial cells treated with aqueous extract of *A. crassna* leaves appeared swelling and distorting after 24 h of incubation. Moreover, as evident by TEM monographs, the extract caused rupture of bacterial cell wall and alteration of bacterial shape compared to control. It has been reported that biofilm formation process consists of two steps, of which the staphylococci first adhere to the foreign-body surface and then accumulate into a complex biofilm structure [23]. The interaction between specific adhesions located on cell wall and extracellular matrix components deposit on the surface is essential for the primary attachment. The destruction of cell wall by the extract is likely to cause bacteria unable to grow and create primary biofilm architecture.

A number of chemicals or plant extracts have shown strong *in vitro* antimicrobial activities with low MIC, however not all of them can be used *in vivo* due to their high toxicity. Because the extract showed relatively high MICs, the acute toxicity of aqueous *A. crassna* leaf extract was investigated to assess its *in vivo* safety and the results showed that all mice did not exhibit abnormal signs of toxicity or deaths after receiving the extract even at high dose (15,000 mg/kg body weight).

## Conclusion

The present study has reported the screening phytochemistry, total phenolic content, and antioxidant activities of the aqueous extract of *A. crassna* leaves. More importantly, the antibacterial activity and inhibitory effect on biofilm formation of the extract against *S. epidermidis* were demonstrated. The postulated underlying mechanism was disruption of bacterial cell wall. In addition, high dose of the extract showed the absence of acute oral toxicity in mice. The data also suggest that *A. crassna* may be a potential source for the discovery of new antibacterial agents against *S. epidermidis* and probably other Gram-positive bacteria as well.

## Competing interests

The authors declared no potential conflicts of interests with respect to the authorship and/or publication of this article.

## Authors’ contributions

SK performed the experiments, analyzed the results and prepared the manuscript. NN, PK, PL and SK provided technical assistance and suggestion. NC supervised the work and corrected the manuscript for publication. All authors read and approved the final manuscript.
